# Incidence of total knee replacement subsequent to intra-articular injection of the anti-inflammatory compound LMWF-5A versus saline: a long-term follow-up study to a randomized controlled trial

**DOI:** 10.1186/s13037-018-0162-4

**Published:** 2018-06-04

**Authors:** John Schwappach, Joseph Schultz, Kristin Salottolo, David Bar-Or

**Affiliations:** 1 Denver Metro Orthopedics, P.C, Englewood, CO 80113 USA; 2 Trauma Research Department, Swedish Medical Center, 501 E. Hampden Ave Rm 4-454, Englewood, CO 80113 USA; 3 Ampio Pharmaceuticals, Inc, 373 Inverness Parkway, Englewood, CO 80112 USA

**Keywords:** Osteoarthritis, Kellgren-Lawrence grade, Total knee replacement

## Abstract

**Background:**

The disease modifying potential of osteoarthritis therapies are of increasing interest, including their effects on delaying total knee replacement (TKR). To date, there have been no studies to determine the effect of LMWF-5A, a novel anti-inflammatory compound derived from human serum albumin, on delaying TKR.

**Methods:**

We evaluated time to TKR three years after patients participated in a randomized trial of three intra-articular injections of LMWF-5A or saline. Patients were contacted via last known phone number and were asked to participate in a short nine item telephone questionnaire; verbal consent was obtained. The primary endpoint was incidence of TKR (%).

**Results:**

In total, 39 of 45 patients responded (87% response rate). The overall incidence of TKR was 38.5% (15/39). TKR rates were higher in patients with more severe osteoarthritis defined by Kellgren-Lawrence grade 4, compared to patients with moderate osteoarthritis defined by Kellgren-Lawrence grade 3 (56% vs. 26%, *p* = 0.06). Overall, there were no differences in TKR rates by treatment arm (39% LMWF-5A vs. 38% saline, *p* = 0.92). In the severe osteoarthritis subset (*n* = 16), treatment with LMWF-5A resulted in a lower incidence of TKR compared to saline vehicle arm (40% vs. 83%, *p* = 0.15). TKR rates were significantly lower with LMWF-5A in patients who responded to treatment (14% with LMWF-5A, vs. 100% with saline, *p* = 0.03).

**Conclusion:**

This study demonstrates significant delays in TKR for patients with severe osteoarthritis treated with LMWF-5A, suggesting that LMWF-5A has the potential to provide structure modifying/preserving therapy in this population.

## Background

Osteoarthritis is a painful, chronic, degenerative, and incurable inflammatory disease. Nearly half of all adults will develop symptomatic osteoarthritis of the knee (OAK) during their lifetime [1]. The course of OAK is progressive to more severe disease, characterized by cartilage loss and increasingly severe clinical manifestations over time [2, 3]. Progression to the most severe form of OAK, defined by Kellgren-Lawrence grade 4 severity (0–4 scale) [4], leaves patients with few treatment options other than surgical interventions including total knee replacement (TKR) surgery. The rate of TKR is anticipated to increase rapidly to over 3.5 million procedures in the U.S. by 2030 [5].

Current osteoarthritis treatments target symptomatic short-term outcomes including pain and function. However, OAK therapies have potential to be disease modifying, including the reduction of cartilage loss and the delay of TKR. Whether OAK therapies can delay TKR is of increasing interest to clinicians, patients, and researchers alike. Very few studies have examined time to TKR or rates of TKR as an outcome parameter, including only three randomized controlled trials (RCTs) [6–8].

LMWF-5A is a novel, non-steroidal, anti-inflammatory compound consisting of the < 5 kilodalton (kDa) ultrafiltrate of 5% human serum albumin (HSA). LMWF-5A is currently in development to provide relief for severe OAK. LMWF-5A has been shown to effectively reduce pain in patients with OAK when administered as an intra-articular injection [9–11]. There have been no in vivo studies determining the effects of LMWF-5A on delaying TKR. In vitro studies suggest aspects of the mechanism of action of LMWF-5A support disease modification [12–15].

The objective of this study was to determine the effect of LMWF-5A on delaying TKR during long-term (three year) follow up of patients participating in a clinical trial evaluating LMWF-5A for the treatment of OAK (AP-007-A) [11].

## Methods

Patients were recruited from the population who participated in the prior clinical trial (AP-007-A). Detailed methods and results of that trial have previously been published [11]. In brief, patients with symptomatic OAK were enrolled between August 1, 2014 and October 19, 2014, with follow-up through October 12, 2015. Patients were randomized 1:1 to three 4 mL intra-articular injections (baseline, week 2, week 4) of either LMWF-5A or saline vehicle control and followed to 20 weeks (primary endpoint), with an exploratory endpoint at 52 weeks to quantitatively measure cartilage thickness change by magnetic resonance imaging (MRI). As reported, LMWF-5A resulted in a significant reduction in pain at 20 weeks compared to saline (64% vs. 40% reduction).

In this IRB-approved follow-up study, patients were followed three years (minimum: 3.1, maximum: 3.3) after treatment with LWMF-5A or saline. Two patients were excluded for mild severity OAK (KL grade 2); the remaining 45 patients were contacted via last known phone number and were asked to participate in a telephone questionnaire. A maximum of three follow-up phone calls were attempted.

During the telephone call, research staff first obtained verbal informed consent and then asked patients a total of a nine questions. The survey included questions asking the patient whether they have received a TKR (q1), time to TKR (q2), reasons for having/not having TKR (q3), past history of analgesic therapies for treating OAK (q4–7), satisfaction with TKR (q8), and overall patient global assessment of disease severity (PGA; q9). Following completion of the study, a copy of the consent form and a $25 gift card was mailed to all consenting patients.

All analyses were performed using SAS 9.3 or later (SAS Institute; Cary, NC). There was no imputation of missing data. Results are presented for all subjects and the subset with severe OAK (KL grade 4). Patients that responded to treatment were also examined, where response to treatment was defined as a 20% (0.5-point) reduction in pain between baseline and week 20 for the primary endpoint of Western Ontario and McMaster Universities Arthritis Index (WOMAC) pain on the 5-point Likert scale. Descriptive statistics were used for all survey questions. The primary endpoint of incidence of TKR (%) was examined with Fisher’s exact tests. The secondary endpoint of time to TKR was examined with Kaplan-Meier survival curves.

## Results

In total, 39 of 45 patients responded (87% response rate); the remaining patients were unreachable. The response rate was similar with LMWF-5A (23/25, 92%) and saline (16/20, 80%). The average time to follow-up was 3.2 years (min: 3.1, max: 3.3 years).

### Survey responses

The overall rate of TKR was 38.5% (15/39). The primary reasons for having TKR were (open-ended): severe osteoarthritis “bone-on-bone” with severe pain (*n* = 6, 40%), severe pain (*n* = 4, 27%), unable to perform activities of daily living (*n* = 3, 20%), no relief from intra-articular injections (*n* = 2, 13%). Reasons patients did not have TKR included: absence of significant pain/mobility limitations (*n* = 13, 54%), currently receiving effective treatment (*n* = 6, 25%), it is contraindicated/not recommended (*n* = 1), fear of operation or its effectiveness (*n* = 1), fear of losing disability insurance (*n* = 1), I do not need it yet/TKR is a “last resort” (*n* = 1), and currently scheduled for TKR (*n* = 1).

In patients who had TKR, 40% (*n* = 6) were receiving knee injections at least a few times each year to treat osteoarthritis, prior to surgery. Among patients who had not had TKR, a similar proportion of patients (37.5%, *n* = 9) reported receiving knee injections, a few times per year (*n* = 5) or less than yearly (*n* = 4).

The majority of responses to the PGA, an overall patient global assessment of disease severity, were very well or well (77%). PGA responses were more favorable in patients who did not have TKR vs. patients with TKR (83% vs. 67% responded very well or well). Still, the majority of patients were satisfied with TKR (87% very satisfied or satisfied).

### Incidence of TKR

**Fig. 1:**
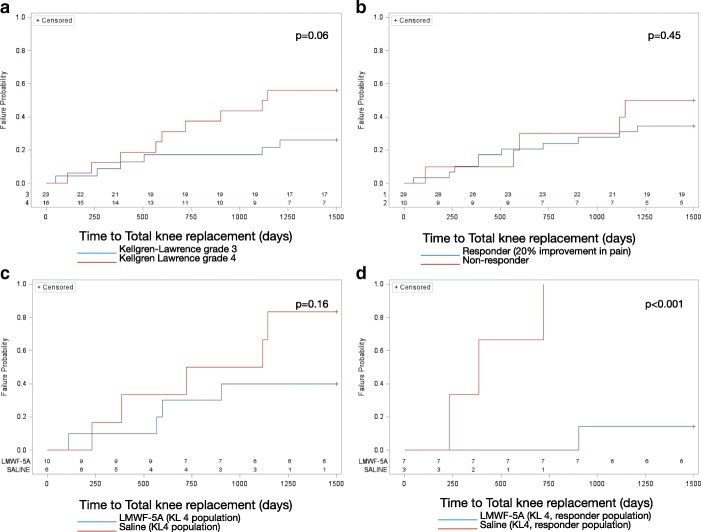
Kaplan-Meier survival curves for total knee replacement. **a** Osteoarthritis severity (Kellgren-Lawrence grade 3 vs. 4); (**b**). Responders to treatment (≥20% improvement in pain by week 20); (**c**). LMWF-5A vs. saline in patients with severe osteoarthritis (Kellgren-Lawrence grade 4); (**d**). LMWF-5A vs. saline in responders to treatment with severe osteoarthritis

Fifteen (39%) patients received TKR on average of 21 months (SD: 13) after the trial commenced. TKR rates were higher in more severe osteoarthritis (KL4: 9/16, 56% vs. KL3: 6/23, 26%, *p* = 0.06). Patients with more severe osteoarthritis also appeared to have TKR more expeditiously (Fig. 1a). TKR rates were similar for responders (10/29, 34%) compared to non-responders (5/10, 50%), *p* = 0.463. There were no differences in the timing of TKR by responder status (Fig. 1b).

Overall, there were no differences in TKR rates by treatment arm (LMWF-5A: 9/23, 39% vs. saline: 6/16, 38%, *p* = 0.92). In the severe KL4 subset (*n* = 16), treatment with LMWF-5A resulted in a lower rate of TKR compared to saline (40% (4/10) vs. 83% (5/6), *p* = 0.15). Patients treated with LMWF-5A also had a longer delay to TKR than saline (Fig. 1c). In patients who responded to treatment during the AP-007-A trial (≥20% reduction in pain), TKR rates were significantly lower in the LMWF-5A arm compared to the saline (14% (1/7) vs. 100% (3/3), *p* = 0.03), with longer delays to TKR (Fig. 1d).

## Discussion

The results of this study are the first to determine the effect of LMWF-5A on delaying TKR. The primary findings suggest treatment with LMWF-5A results in significant delay in TKR for patients with severe OAK. This result agrees with previous findings that the treatment effect of LMWF-5A is greatest in patients with more severe disease [9, 10].

Federal Drug Administration (FDA) guidelines propose that trials designed to evaluate delays in structural progression measure joint space narrowing [16]. Time to TKR may also be an acceptable outcome. Most studies examining time to TKR have been retrospective case series. There have only been three RCTs to examine time to TKR in patients with OAK. Raynauld et al. performed a post hoc follow-up four years after a twelve month RCT, with an 81% response rate among 57 patients; the rate of TKR was non-significantly lower in patients randomized to chondroitin sulfate vs. saline control (31% vs. 69%) [8]. Bruyere et al. evaluated patients participating in two previous RCTs who received at least one year of glucosamine sulphate (response rate 81%); the rate of TKR was significantly lower with glucosamine sulphate compared to placebo (6.3% vs. 14.5%) [6]. Blanco et al. examined time to TKR among 52 patients with severe OAK randomized 1:1 to hyaluronic acid or saline; the rate of TKR was non-significantly lower with hyaluronic acid compared to saline (64% vs. 87%) [7]. Only the Blanco study excluded lower severity OAK; compared to that study, our rate of TKR was similar for saline arms (87% and 83%, respectively), with a lower rate of TKR with LMWF-5A than hyaluronic acid (40% and 64%, respectively).

The findings from this study support the MRI analysis of study AP-007-A that demonstrated potential cartilage preservation with LMWF-5A compared to saline, representing potential for disease modification. There were 37 patients with MRI data at baseline and at week 52, including 20 patients with medial (*n* = 10) or lateral disease (*n* = 10) and the remaining (*n* = 17) with either no denudement or symmetrical disease. Patients treated with LMWF-5A had less cartilage thickness loss than patients treated with saline in all 6 pre-specified anatomically defined medial sub regions (medial disease), and in 5 of 6 lateral sub regions (lateral disease). LMWF-5A also showed increased cartilage thickness in 2 lateral sub regions.

There are limitations. First, this was a post hoc analysis that was not powered to detect differences in TKR rates between treatment groups. A prospective RCT with incidence of TKR as the primary outcome would need to be conducted to confirm these findings. Second, not all patients were reachable; 6 patients had disconnected phone numbers. Third, respondents were asked “Have you had a total knee replacement in your study knee?”. It is possible that subjects with a partial knee replacement might have responded negatively. Lastly, there are multiple factors that may have influenced the decision to have TKR, including current use of other pharmacologic interventions. We collected information on use of intra-articular injections and they were reported in a similar occurrence in the TKR group (prior to surgery) and the non-TKR group (40% vs. 37.5%), but there remains the risk of recall bias.

## Conclusion

This study demonstrated delays to TKR among patients with severe osteoarthritis treated with LMWF-5A compared to saline, while showing no differences in patients with moderate osteoarthritis. These results support the in vitro work suggesting the effect of LMWF-5A is demonstrated with severe disease, and has the potential to provide structure modifying/preserving therapy in this population.

## Abbreviations

HSA: Human serum albumin
IRB: Institutional review board
KDa: Kilodalton
KL: Kellgren Lawrence
LMWF-5A: Low Molecular Weight Fraction of 5% human serum Albumin
MRI: Magnetic resonance imaging
OAK: Osteoarthritis of the knee
PGA: Patient global assessment
RCT: Randomized controlled trial
TKR: Total knee replacement
WOMAC: Western Ontario and McMaster Universities Arthritis Index
